# Paired metagenomic and chemical evaluation of aflatoxin-contaminated dog kibble

**DOI:** 10.3389/fvets.2024.1374839

**Published:** 2024-04-11

**Authors:** Andrea Ottesen, Brandon Kocurek, Elizabeth Reed, Seth Commichaux, Mark Mammel, Padmini Ramachandran, Patrick McDermott, Brenna M. Flannery, Errol Strain

**Affiliations:** ^1^Center for Veterinary Medicine (CVM), U.S. Food and Drug Administration, Laurel, MD, United States; ^2^Center for Food Safety and Applied Nutrition (CFSAN), U.S. Food and Drug Administration, College Park, MD, United States

**Keywords:** metagenome, metagenomic, chemical, aflatoxin, kibble, *Aspergillus*

## Abstract

**Introduction:**

Identification of chemical toxins from complex or highly processed foods can present ‘needle in the haystack’ challenges for chemists. Metagenomic data can be used to guide chemical toxicity evaluations by providing DNA-based description of the wholistic composition (eukaryotic, bacterial, protozoal, viral, and antimicrobial resistance) of foods suspected to harbor toxins, allergens, or pathogens. This type of information can focus chemistry-based diagnostics, improve hazard characterization and risk assessment, and address data gaps. Additionally, there is increasing recognition that simultaneously co-occurring mycotoxins, either from single or multiple species, can impact dietary toxicity exposure. Metagenomic data provides a way to address data gaps related to co-occurrence of multiple fungal species.

**Methods:**

Paired metagenomic and chemical data were used to evaluate aflatoxin-contaminated kibble with known levels of specific mycotoxins. Kibble was ground to a fine powder for both chemical and molecular analyses. Chemical analyses were performed with Liquid Chromatography Mass Spectrometry (LCMS) and according to the AOAC Official method 2005.08: Aflatoxins in Corn, Raw Peanuts, and Peanut Butter using Liquid Chromatography with Post-Column Photochemical Derivatization. Metagenomes were created from DNA extracted from ground kibble and sequenced on an Illumina NextSeq 2000 with an average sequence depth of 180 million reads per replicate.

**Results and discussion:**

Metagenomic data demonstrated that the abundance of DNA from putative aflatoxigenic Aspergillus spp. correlated with the levels of aflatoxin quantified by LCMS. Metagenomic data also identified an expansive range of co-occurring fungal taxa which may produce additional mycotoxins. DNA data paired with chemical data provides a novel modality to address current data gaps surrounding dietary mycotoxin exposure, toxigenic fungal taxonomy, and mycotoxins of emerging concern.

## Introduction

The evaluation of new methodological approaches to identify microbiological and chemical hazards in human and animal foods is a central focus of FDA research (1). Advancing science to improve regulatory policy is a central dogma underpinning the Agency’s public health mission. Currently, genomic and metagenomic data are used for food safety applications such as source tracking pathogens (2, 3), identification of adulterants, contaminants, toxins, and allergens (4), and detection and prediction of antimicrobial resistance (AMR) (5). A new frontier of utility for metagenomic data includes support for chemical toxicity evaluations through provision of wholistic DNA-based profiles of macro (plant, animal, and insect) and micro (bacterial, fungal, viral, and AMR) components of both unprocessed and highly processed human and animal foods.

Molecular tools have facilitated a paradigm shift in our understanding of the ecology of pathogenicity associated with human and animal foods (6). A similar trend in mycotoxin risk assessment has evolved to consider simultaneous co-exposure to diverse mycotoxins (7). Mycotoxins are secondary metabolites produced by fungi. There are hundreds of different moieties produced by single and/or multiple species. It is rare for a single mycotoxin to exist in any crop or food due to the complex biodiversity of agricultural ecologies (8). Reported fungal species for corn alone include *Fusarium proliferatum, Trichoderma gamsii, T. longibrachiatum, Penicillium oxalicum, P. aurantiogriseum, P. polonicum, Bipolaris zeicola, Sarocladium zeae, Chaetomium murorum, Botrytrichum murorum, Cladosporium cladosporioides, C. sphaerospermum, Aspergillus niger, A. flavus, Alternaria alternata*, and *Rhizopus microsporus* (9), at least half of which are known to produce toxins of significance to human and animal health.

Risk assessment of mycotoxins in foods has, to date, primarily focused on a small number of important toxins with critical adverse effects, without extensive consideration of co-exposure to multiple compounds (7), despite the fact that the presence of multiple mycotoxins is more common than the presence of a single mycotoxin. Recent studies of animal feed in Europe found that 75–100% of the examined feed contained more than one mycotoxin (10). Exposure to a single toxin or multiple mycotoxins can result in adverse effects (7). While much work has been done to describe mycotoxin co-exposure, there is still more to learn about dietary toxicities associated with co-occurring mycotoxins.

*Aspergillus flavus* and *A. parasiticus* are two of the most traditionally recognized aflatoxigenic species in pre- and post-harvest commodities, but there are numerous other aflatoxin-producing species of *Aspergillus,* and it has even been proposed that species of *Fusarium, Penicillium, Claviceps,* and *Alternaria* may produce aflatoxins (11, 12). The International Agency for Research on Cancer (IARC) has classified aflatoxins as the most potent natural carcinogens known to humankind and they are estimated to contaminate 25% of crops worldwide (13). Many commonly used ingredients for animal food, such as corn, wheat, and rice, are susceptible to contamination by mycotoxins.

Whole genome sequencing (WGS) and metagenomic sequencing (MGS) have been used to describe how pathogens and toxins become associated with human and animal foods (6, 14–19), but a new frontier of integrated chemical and metagenomic data is on the horizon for modernized evaluation of chemical toxins in foods. Here we used MGS data to describe how the taxonomic abundance (amount of DNA) of putative aflatoxigenic fungal species, *Aspergillus*, correlated with levels of aflatoxin quantified by Liquid Chromatography Mass Spectrometry (LCMS). Two levels of aflatoxin-contaminated kibble, one at 15 ppb and one at 522 ppb, were shown to correlate with low and higher abundance (respectively) of *Aspergillus* DNA. Additionally, we demonstrated that a wide range of species that may produce additional mycotoxins could be identified by metagenomic data, to better address current data gaps and modernize mycotoxin risk assessment.

## Materials and methods

### Mycotoxin evaluation

Low (15 ppb) and high (522 ppb) levels of aflatoxin-contaminated kibble were measured according to standard operating procedures of the Plant Industries Division of the Missouri Department of Agriculture, which uses the AOAC Official method 2005.08: Aflatoxins in Corn, Raw Peanuts, and Peanut Butter (Liquid chromatography with Post-Column Photochemical Derivatization). Samples were subsequently sent to the Center for Veterinary Medicine, FDA for metagenomic analysis. Kibble measuring 15 ppb aflatoxin were considered “low” and kibble measuring 522 ppb were considered “high” level aflatoxin samples for the purposes of this study. Aflatoxin at 15 ppb in kibble is below the FDA action level for aflatoxins of 20 ppb in pet food (20). An additional LCMS Multiple Mycotoxin evaluation was conducted by a third-party Feed Evaluation Lab (Cumberland Valley Analytical Services, Zullinger, PA). Mycotoxins evaluated included aflatoxin, fumonisin, ochratoxin A, and zearalenone in control and contaminated kibble.

“Control” samples were taken from the exact same brand of dog food but different lot number (not part of the recalled aflatoxin-contaminated kibble) to represent a baseline. Kibble ingredients listed chicken by-product meal, corn, wheat, meat meal, rice bran, chicken fat, dried beet pulp, whitefish meal, flaxseed, salt, potassium chloride, choline chloride, vitamins, and minerals. The label described a composition of at least 26% protein, 15% fat with maximum fiber at 6%, moisture at 10%, and 3,645 kcal/kg.

### Metagenome preparation

Three 2.5 g portions of each level of aflatoxin-contaminated kibble and control were ground to a fine powder in a Qiagen Tissue Lyser II at 30 Hz (1800 oscillations per minute) for 1.3 min per sample. Replicates of 100 mg of powder from the pooled mixture (7.5 gram) were used for DNA extraction. DNA extraction was performed using the Zymo High Molecular Weight DNA extraction kit according to the manufacturer’s specifications including an extra lysing step using PBS and lysozyme (21). Libraries of DNA were created using the Illumina DNA Library Prep Kit according to the manufacturer’s specified protocols and sequencing was performed on a NextSeq 2000 using a high-throughput kit according to previously described methods (22). An additional NextSeq 2000 high-throughput sequencing run was performed with only two replicates of low- and high-level samples to achieve a sequencing depth between 100 and 250 million reads per replicate to evaluate how increased read depth impacted incidence of key species.

### Bioinformatic analyses

Sequence data were screened for quality metrics using Trimmomatic (23) and analyzed using in-house FDA pipelines and databases as previously described (22). Determination of bacterial and fungal composition from shotgun sequencing was conducted using custom C++ programs developed to compile a *k*-mer signature database containing multiple unique 30 bp sequences per species and then identify each read in the input file using the 30 bp probes. For each bacterial or fungal species or subspecies, each non-duplicated 30-mer from a reference whole genome sequence was placed into a database. Any *k*-mers not found in at least 2/3 of a set of additional genome sequences of the same species were removed and *k*-mers found in genomes of other species were removed. Normalization was performed to correct for bias due to differing number of *k*-mers per database entry and results were tabulated as percent of identified reads (contribution to the microbial population of identified species) for each database entry. Results can be expressed by raw hits or as relative abundance. Both are useful when detecting low abundance organisms in complex metagenomes. Annotation was also done using the Cosmos ID cloud-based application (CosmosID Metagenomics Cloud, app.cosmosid.com, CosmosID Inc., www.cosmosid.com) with Fungal Database Version 1.2 (24). LEfSe (Linear discriminant analysis effect size) by the Huttenhower biobakery^1^ (25) was calculated in the COSMOSID application. LEfSe (Linear discriminant analysis effect size) is an algorithm used for biological biomarker discovery. Features such as genes, pathways, or taxa were identified for each treatment and the non-parametric factorial Kruskal-Wallis (KW) sum-rank test (26) was used to identify significant differential abundance of specific features between treatments. Linear Discriminant Analysis (LDA) was used to estimate the effect size of each differentially abundant feature and rank the feature accordingly. For the bar chart presented in the results section, the LDA ranged from 2.9 to 5.27 and the *p* value ranged from 0.022 to 0.035. Sequence data annotations were visualized using graphs created by the R Tidyverse package.^2^

### Mycotoxin pathway identification

Metagenomic reads were mapped with Diamond (v2.0.5) (27) BLASTX (≥95% identity and those ≥90% read coverage) to a database of genes involved in mycotoxin biosynthetic pathways: aflatoxin, deoxynivalenol, nivalenol, ochratoxin, patulin, sterigmatocystin, T2_toxin, and tenuazonic_acid. Genes were identified and downloaded from the MetaCyc database (28).^3^ Identity of reads aligning to mycotoxin genes was confirmed by BLASTX, aligning them to the NCBI nr database online.

## Results

### Kibble ingredients identified by metagenomic sequencing

To describe composition and relative abundance of ingredients, metagenomic data were created for three replicates of control, three replicates of low-concentration (15 ppb), and three replicates of high-concentration (522 ppb) aflatoxin-contaminated kibble. An average of 182 million sequences per replicate were used in downstream analyses. The relative abundance of macro ingredients [annotated by mitochondrial DNA (29) and occurring at greater than 1% of normalized data] included *Zea* (corn), *Gallus* (chicken), *Triticum* (wheat), Soya, (soybean), *Bos* (cow, ox, bull, yak, cattle), and yeast. Further refinement of species annotations described *Z. mays, G. gallus*, *G. gallus ssp. spadiceus, T. aestivum*, *B. taurus*, several species of *Saccharomyces* (*cerevisiae, pastorianus*, and *pastorianus* Weihenstephan), and *Fusarium verticillioides.*

### *Aspergillus* species in kibble

**Figure 1:**
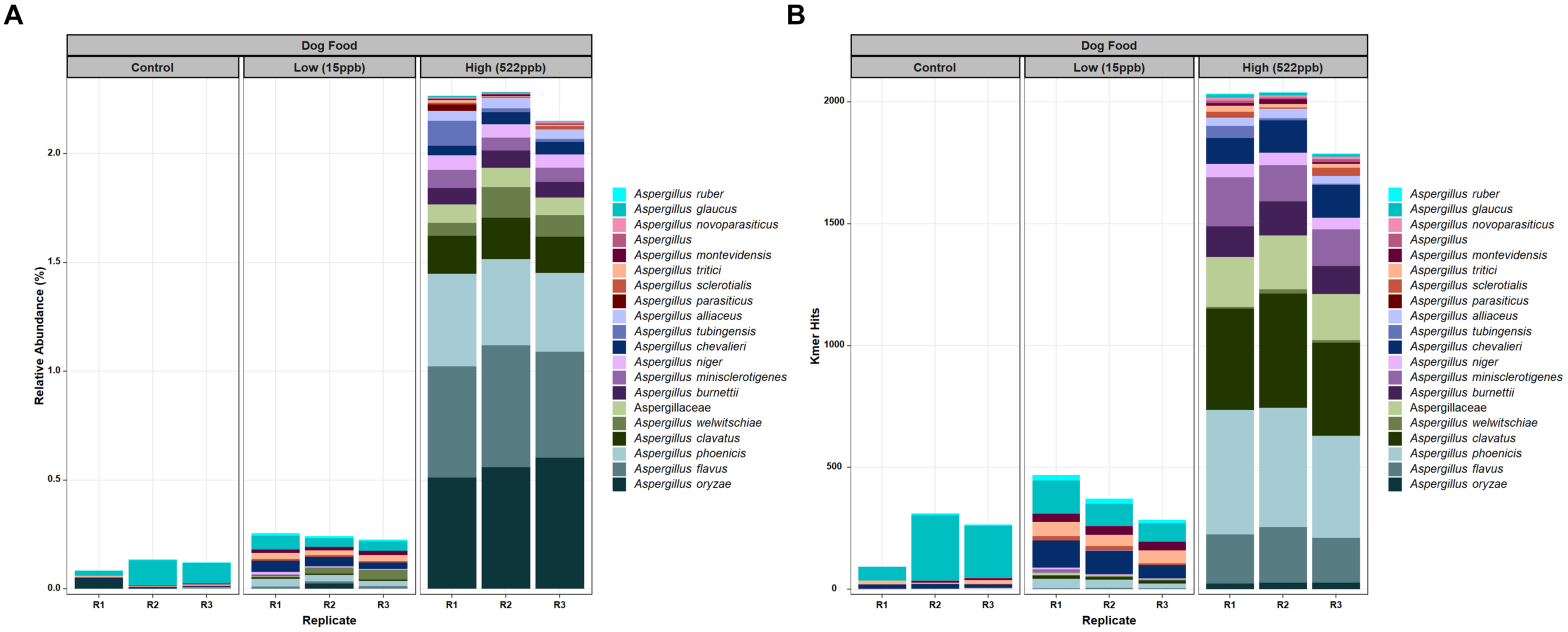
*Aspergillus* species in control, low, and high level aflatoxin contaminated kibble. **(A)** Relative abundance of the top twenty *Aspergillus* species and **(B)** Raw (no normalization) k-mer hits to *Aspergillus* species. *Aspergillus* was annotated using an in-house FDA fungal database as described in Methods.

Evaluation of the metagenomic data from control, low-, and high-level aflatoxin-containing kibble correlated with relative abundance of DNA of *Aspergillus* species, i.e., the low-level aflatoxin-contaminated kibble had a low abundance of DNA from *Aspergillus* species and the high-level kibble had a higher relative abundance of *Aspergillus* species. The incidence of Aspergillus or any other taxon can be expressed by raw hits to the genome of interest or as relative abundance after normalization by k-mer count per organism and number of sequence reads. Almost no *Aspergillus* DNA was detected in controls (Figure 1). The *Aspergillus* species identified in control kibble was predominantly *A. glaucus*. Figure 1A shows the top twenty most abundant *Aspergillus* species identified in control, low- (15 ppb), and high- (522 ppb) levels of aflatoxin-contaminated dog kibble using relative abundance normalization. Figure 1B shows the raw k-mer hits to the top 20 *Aspergillus* species seen in the metagenomes. Both approaches, relative abundance and raw k-mer hits, are useful when attempting to detect low abundance DNAs from low abundance organisms. There was an extensive taxonomic range of *Aspergillus* species identified in the kibble. Production of aflatoxin may have been associated with a single species or potentially multiple species. *Aspergillus oryzae*, *flavus,* and *phoenicis* were the most abundant species observed in high-level (522 ppb) aflatoxin-contaminated kibble. *Aspergillus flavus* and *A. parasiticus* are two of the most traditionally recognized producers of aflatoxin in pre- and post-harvest commodities (12). These species likely played a role in aflatoxin production in the kibble as *A. oryzae* and *A. phoenicis* are not known to produce aflatoxins (30, 31).

### Additional mycotoxin detection and associated species

While aflatoxin B1 was the primary focus of the chemical evaluation of the kibble due to its acute toxicity, concentrations of fumonisins (B1,B2,B3), and ochratoxin A were also detected by LCMS (Table 1).

**Table 1:** Chemical concentrations of mycotoxins in low and high levels of aflatoxin-contaminated dog kibble.

Toxin	Low aflatoxin kibble	High aflatoxin kibble
Aflatoxin B1	15.5 ppb	522.4 ppb
Aflatoxin B2	0	27.0 ppb
Aflatoxins B1 + B2	15.5 ppb	549.4 ppb
Zearalenone	0	0
Ochratoxin A	0	23.1 ppb
Fumonisin B1	0.9 ppm	1.5 ppm
Fumonisin B2	0.2 ppm	0.4 ppm
Fumonisin B3	0.1 ppm	0.2 ppm
Fumonisins (B1 + B2 + B3)	1.2 ppm	2.1 ppm

**Figure 2:**
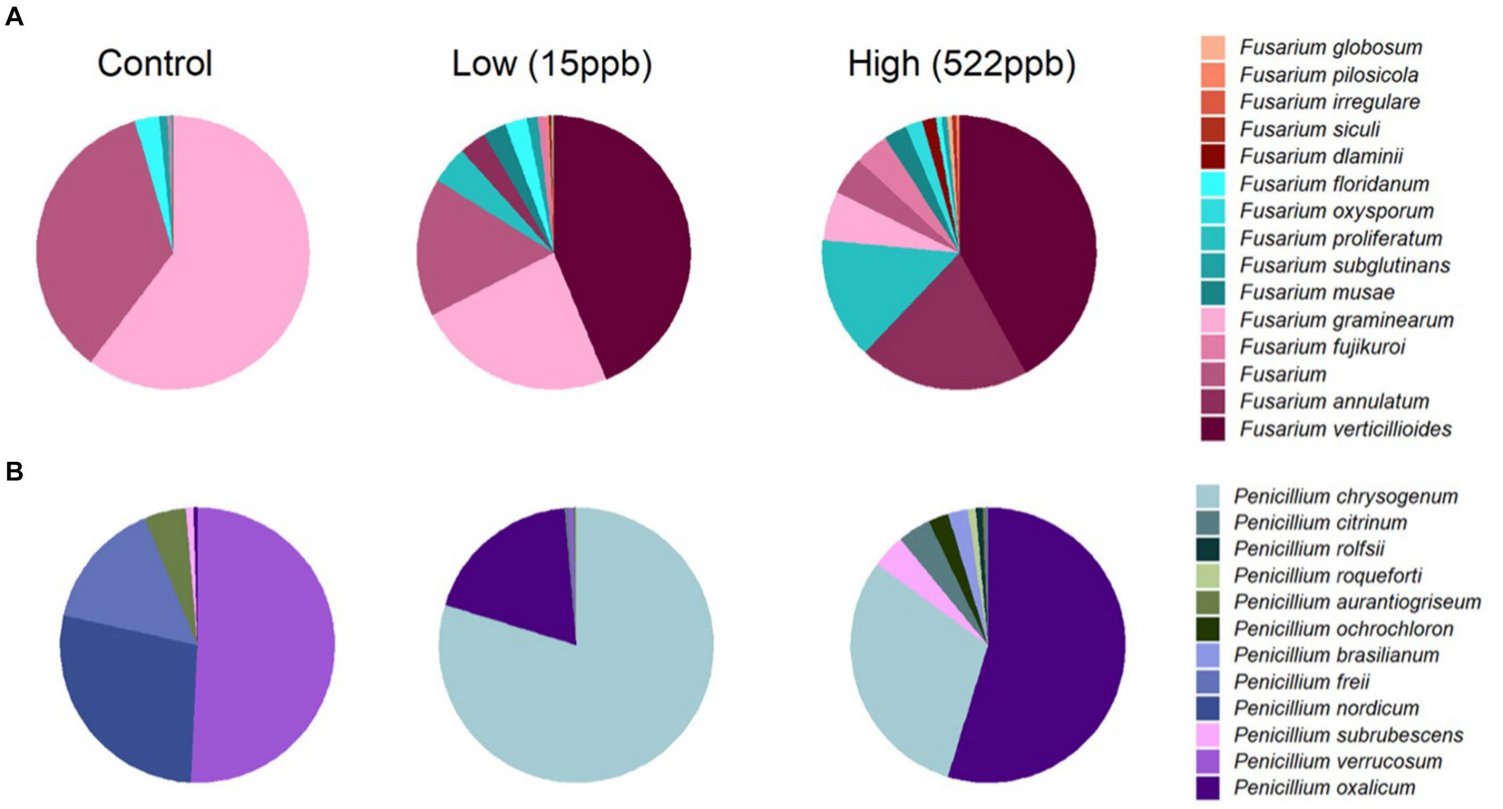
Relative abundance of *Fusarium* and *Penicillium* species in control, low, and high levels of aflatoxin contaminated kibble. **(A)** Shows the average relative abundance of *Fusarium* species from three replicates of each kibble type, (control, low, and high level aflatoxin contamination) annotated using an in house FDA fungal database developed for metagenomic data. **(B)** Shows the average relative abundance of *Penicillium* species in each kibble type. Contaminated kibble had a distinctive incidence of *Fusarium verticillioides*, *Penicillium oxalicum* and *P. chrysogenum*, not seen in control kibble.

*Fusarium verticillioides* is reported to produce fumonisins and zearalenone and was observed in both low and high samples of kibble but not in controls (Figure 2A). Additional *Fusarium* species, including *F. annulatum, proliferatum, dlaminii, siculi, irregulare, pilosicola,* and *globosum*, were primarily associated with contaminated kibble and were not observed in controls (Figure 2A). Deoxynivalenol is also produced by Fusarium species but was not measured in this study.

*Penicillium* spp. Are reported to produce Ochratoxin A and patulin (32). *Penicillium oxalicum*, *subrubescens, brasilianum, ochrochloron, roqueforti,* and *citrinum* were primarily observed in contaminated samples whereas *P. verrucosum*, *nordicum*, and *freii* were the most abundant species observed in controls (Figure 2B). It is possible that *P. oxalicum* or *P. subrescens* played a role in the observed ochratoxin levels (23 ppb) in ‘high’ samples. However, *P. oxalicum* was also observed in low-level samples for which no ochratoxin was quantified, so perhaps other genera that were differentially enriched in high-level samples were responsible for the ochratoxin observed by LCMS (Table 1).

### Biomarker discovery

**Figure 3:**
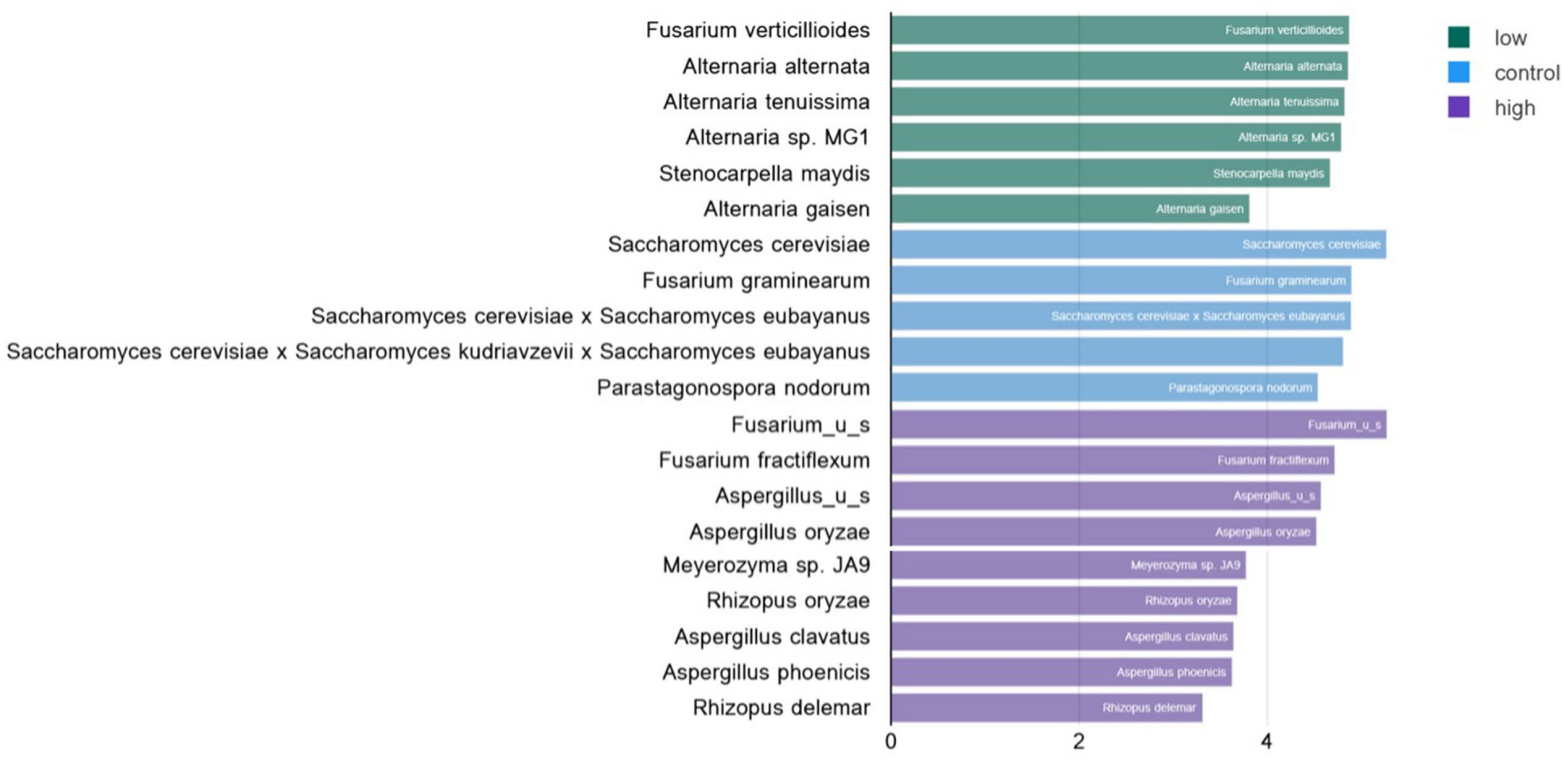
Linear discriminate analysis (LDA) of significant differential abundance of fungal species in low, control, and high level aflatoxin contaminated dog kibble. Using the LefSE tool available in the COSOMOSID annotation pipeline taxa that were significantly enriched in each type of kibble (control, low, high) were described. The LDA threshold spanned 2.9 – 5.27 with a *p* value range of 0.022–0.035. This approach provides biomarkers that may correlate with risk of aflatoxin contamination or that may correlate with safe or low risk kibble.

Linear discriminant analysis of fungal species in low- and high-level contaminated kibble was calculated using the Huttenhowever biobakery tools (25) available in the COSMOSID analysis pipeline (CosmosID Metagenomics Cloud, app.cosmosid.com, CosmosID Inc., www.cosmosid.com). This tool functions as a biomarker discovery tool, comparing differential abundance of taxonomy or functional genes in different treatment groups. Used with fungal taxonomy for control, low-, and high-level aflatoxin contaminated kibble, it was clear that high-level kibble contained a greater abundance of *Aspergillus* species than low-level kibble (Figure 3). Interestingly, control kibble had significantly more *Saccharomyces* species than the high-level kibble (*p* < 0.022 to 0.035). It is common practice to add yeast to ingredients that contain aflatoxin. Yeast species have been shown to bind aflatoxin B and, thus, reduce the toxic impact of consumption of aflatoxin-contaminated food (33). This approach has been shown to provide a protective effect to broiler chickens consuming aflatoxin B1-contaminated food (34). Yeast was listed as an ingredient in all dog kibble.

**Figure 4:**
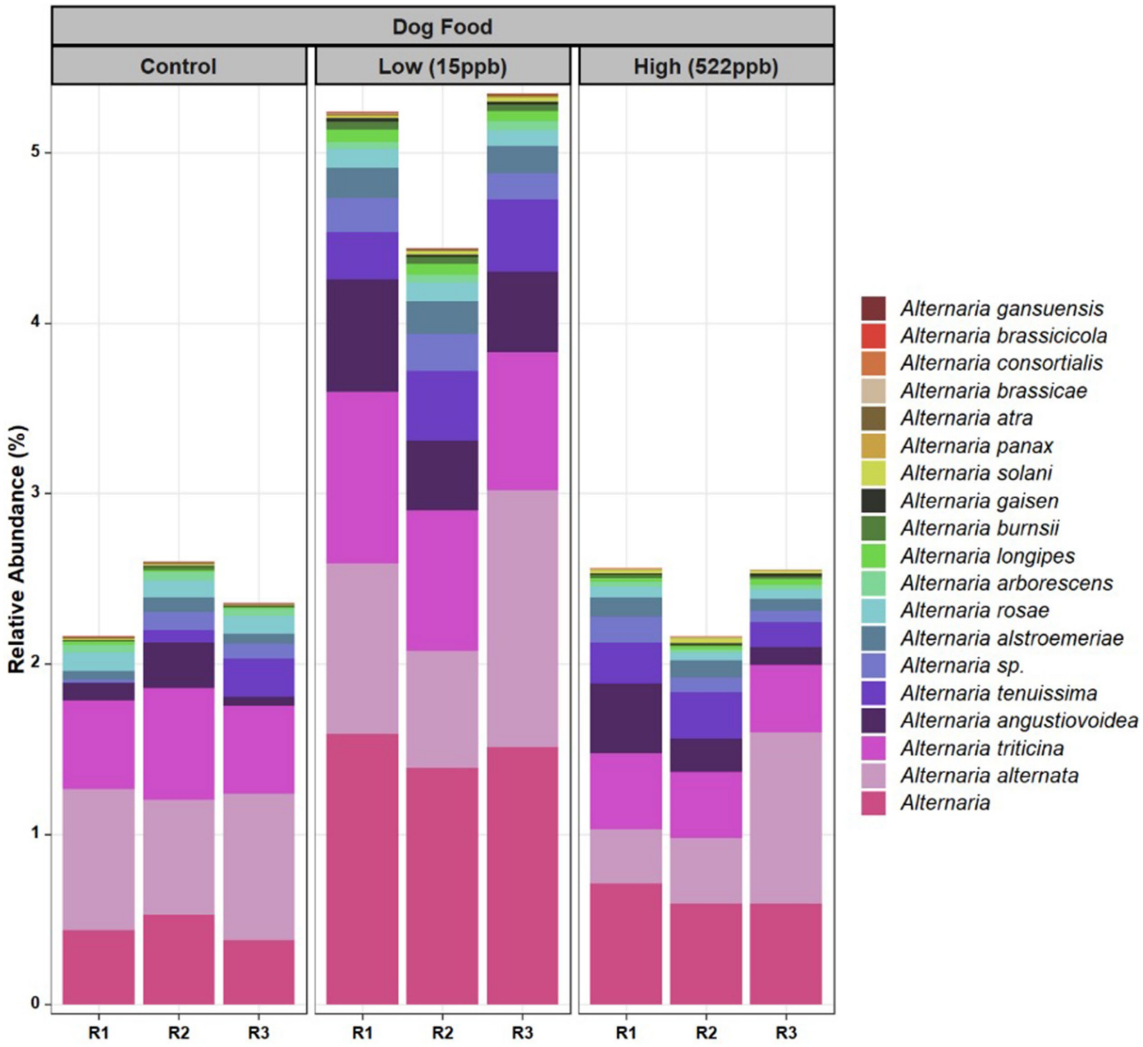
Species of *Alternaria* identified in control, low, and high level aflatoxin contaminated kibble. Species of *Alternaria* observed in the control, low, and high level aflatoxin contaminated kibble are shown for each of three replicates. For the most part, similar species were observed across all kibble types, however a greater relative abundance was observed in low level samples.

Additionally, a greater relative abundance of *Alternaria* species were observed in low-level aflatoxin-contaminated kibble. While *Alternaria* species were observed in metagenomic data, *Alternaria*-associated toxins (altenuene, alternariol, alternariol monomethylether, tentoxin, and tenuazonic acid) were not measured in this study. Figure 4 provides a breakdown of the species of *Alternaria* across each kibble type.

**Figure 5:**
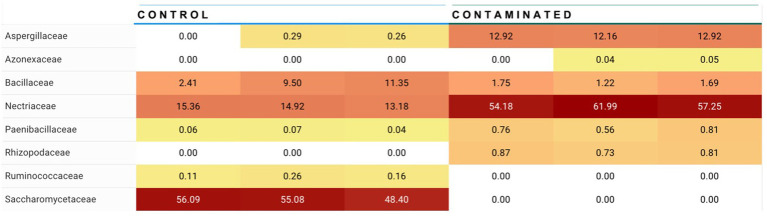
Summary of differential abundance at fungal family level. Families *Apergillaceae* (containing *Aspergillus* genera) and *Nectriaceae* (containing *Fusarium* genera) were observed at significantly higher abundance in contaminated kibble (high level aflatoxin) than in control kibble, and *Saccharomycetaceae* (containing *Saccharomyces* genera i.e., yeast) are at significantly higher abundance in control kibble contrasted to contaminated kibble.

For *Alternaria*, species composition from control to contaminated kibble was more stable than what was observed for genera like *Aspergillus, Penicillium,* and *Fusarium* (Figures 1, 2). Fingerprints of differential abundance of fungal taxa (Figure 5) could potentially be used to quickly detect lots of feed or ingredients that may be at higher risk of aflatoxins or other mycotoxin exposure. When *Saccharomyces* species (used to bind aflatoxin) have been depleted, there is likely a greater risk of toxicity exposure.

### Identification of functional genes in mycotoxin pathways

Beyond identification of the taxonomic structure of kibble, metagenomic data also described functional genes involved in mycotoxin production and thus provided further confirmation of mycotoxin pathways such as deoxynivalenol, nivalenol, and aflatoxin. Not surprisingly, the most numerous hits to genes involved in the aflatoxin production pathway were seen in the high-level aflatoxin-contaminated samples (Table 2). The doublet and singleton hits to nivalenol (NIV) and deoxynivalenol (DON) in control samples may be indicative of low levels of NIV or DON in these samples.

**Table 2:** Hits to genes in deoxynivalenol, nivalenol, and aflatoxin pathways in metagenomic data of kibble.

ID	Pathways	Genes	Confirmed
Control	deoxynivalenol	TRI8	1
Low15ppb	nivalenol	TRI1	2
High522ppb	aflatoxin	AFLC, AFLK, AFLV, AFLL, AFLP, ORDA, AFLQ, AFLW, AFLX, AFLY, AFLS	56

The number of hits to genes in mycotoxin production pathways for deoxynivalenol, nivalenol, and aflatoxin is show in the “confirmed” column. Hits were blasted to NCBI to confirm gene identification.

### Additional interesting observations

*Stenocarpella maydis,* another potential toxin producer, was observed in the contaminated kibble samples but not in controls. *Stenocarpella* was identified as a biomarker for low-level aflatoxin samples where it was significantly enriched (Figure 3). Toxic metabolites including diplodiatoxin, chaetoglobosins K and L, and (all-E)-trideca-4,6,10,12-tetraene-2,8-diol associated with *Stenocarpella* are not commonly evaluated and simple ELISA or other HPLC based tests are not readily available.

### Bacterial species in metagenomes of dog kibble

**Figure 6:**
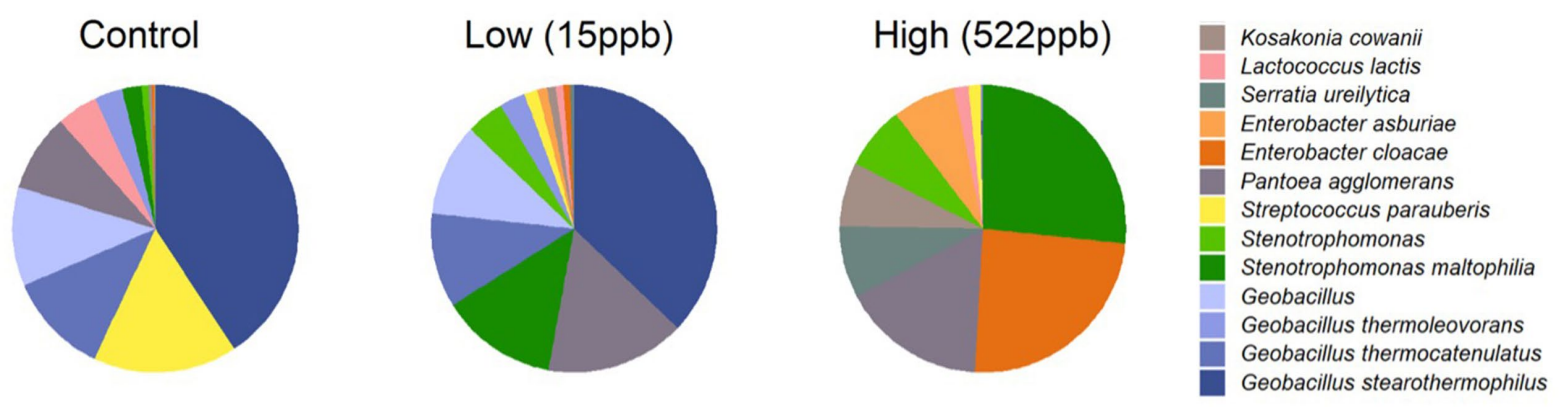
Bacterial species in control, low, and high level aflatoxin contaminated kibble. Bacterial taxa from control, low, and high level-aflatoxin contaminated dog kibble are shown here. The high level kibble had a strong incidence of *Enterobacter cloaceae*. *Stenotrophomonas* which has been described as an emerging (at times multi-drug resistant) global pathogen, was also seen in the high level kibble.

Bacterial profiles for controls and aflatoxin-contaminated dog kibble are shown in Figure 6. Three replicates of control, low-, and high-level aflatoxin-contaminated dog kibble were averaged and taxa occurring at greater than 3% of total data were graphed to summarize bacterial features of each kibble. *Enterobacter, Serratia, and Kosakonia genera* were observed in toxin-contaminated kibble and not in control kibble.

### Antimicrobial resistance in kibble

Control kibble had different antimicrobial resistance genes than those observed in aflatoxin-contaminated kibble. This is most likely driven by the different bacteria that were present in contaminated samples contrasted to controls. Genes involved in macrolide resistance were seen in contaminated kibble as well as beta-lactam genes *bla* R1 and *bla* I, which influence expression of *bla*Z and penicillin binding protein 2a (PBP 2a) and contribute to methicillin resistance (35, 36). The *bla* 1 gene was observed in control samples, typically associated with ampicillin resistance.

## Discussion

A primary mission of the Center of Veterinary Medicine (CVM) is to protect animal health by ensuring the safety of animal food. The application of metagenomic data to complement toxicity identification evaluations (TIEs) presented here is a novel methodological approach to support the mission of safeguarding food. Metagenomic data provides comprehensive DNA-based identification of all eukaryotic (insect, plant, and animal) and prokaryotic (bacteria, fungi, and protist) constituents of any food, including adulterants, allergens, pathogens, antimicrobial resistance determinants and biomarkers. This type of information can help chemists focus search parameters when unknown chemical toxins have caused foodborne illness.

In this work, we demonstrated that metagenomic data from kibble with low and high levels of aflatoxin contamination correlated with the incidence and abundance of DNA from *Aspergillus* species. This observation supports the hypothesis that *Aspergillus* species played a role in the aflatoxin production that led to the levels seen in the high kibble. Additionally, the genes in the aflatoxin production pathway were only identified in high-level kibble, supporting the utility of metagenomic data to identify aflatoxin in food. Statistically significant biomarkers associated with high-level aflatoxin included four differentially enriched *Aspergillus* taxa and distinctive *Rhizopus* and *Fusarium* taxa. Statistically significant biomarkers associated with the control kibble included four differentially enriched *Saccharomyce*s taxa, which, as previously described, is often added to foods to bind aflatoxin and make food safer for consumption. Metagenomic data could serve as a critical control point assessment to identify robust populations of *Saccharomyces* in kibble before it is sold to consumers. Biomarkers may also inform on regional and/or temporal origins of ingredients (31). Understanding exactly how DNA biomarkers perform as predictors of toxicity will require future validation work but these preliminary results are very promising.

Metagenomic data used in concert with chemical profiling is not only useful to detect contamination by known aflatoxigenic species but also to describe unknown species of mycotoxigenic fungi in human and animal foods. The FDA currently monitors for aflatoxins, fumonisins, vomitoxin, zearalenone, and ochratoxin A in human and animal food using single target methods (37, 38), although a multi-mycotoxin surveillance approach has recently been initiated by the FDA for infant and toddler foods (39). Metagenomics provides a non-targeted approach that can describe mycotoxigenic fungi that may produce toxins not currently monitored by the FDA and data could signal that additional mycotoxin testing may be needed to accurately characterize hazards and assess risk.

The metagenomic data described here identified multiple *Penicillium* species, which can produce toxins such as brevianamid A, citreoviridin, citrinin, cyclopiazonic acid, fumitremorgin B, griseofulvin, luteoskyrin, penicillic acid, penitrem A, PR-toxin, roquefortine, rugulosin, verrucosidin, verruculogen, viridicarumtoxin, and xanthomegnin (40). Concordant chemical testing could establish if these compounds were present or not. Metagenomic data also identified *Stenocarpella maydis, Rhizopus delemar*, *and R. oryzae* in contaminated kibble and not in control kibble. *Stenocarpella maydis* is a fungal pathogen of corn and its toxic metabolites include diplodiatoxin, chaetoglobosins K and L, and (all-E)-trideca-4,6,10,12-tetraene-2,8-diol (41, 42). Synonyms for *S. maydis* include *Diplodia zeae, Diplodia maydis, Sphaeria maydis, S. zeae, Macrodiplodia zeae,* and *Dothiora zeae*, which have been linked to diplodia toxicity (diplodiosis). Diplodiosis is characterized by muscle tremors, incoordination, ataxic hindquarters, paralysis, and death of cattle, sheep, rats, and ducklings, with reports of cattle mortalities dating back to 1919 (43).

Using a limited case study with recalled kibble with two known levels of aflatoxin (15 ppb and 522 ppb), we demonstrated that DNA of putative aflatoxin producers was differentially enriched in low- and high-level kibble (i.e., comparatively high amounts of *Aspergillus* DNA were seen in the high-level aflatoxin contaminated kibble contrasted to the low-level). We also documented the aflatoxin production pathway by the identification of genes in that pathway which were also only seen in the high-level aflatoxin kibble DNA. The taxonomic identification of additional potential toxin producers could serve to guide additional chemical testing to better understand if other toxins were present in the food and which species may have produced them. Metagenomic data can describe the total composition (genus, species, subspecies, and even serovars and varieties) of plants, animals, insects, bacteria, fungi, viruses, plasmids, and antimicrobial resistance. Paired chemical and MGS data is an exciting frontier that will undoubtedly provide valuable information to underpin modernized risk assessment for human and animal foods.

## Data availability statement

The datasets presented in this study can be found in online repositories. The names of the repository/repositories and accession number(s) can be found at: https://www.ncbi.nlm.nih.gov/bioproject/PRJNA1062328/.

## Author contributions

AO: Writing – original draft, Visualization, Supervision, Project administration, Methodology, Investigation, Formal analysis, Data curation, Conceptualization. BK: Writing – review & editing, Visualization, Project administration, Methodology, Formal analysis, Data curation. ER: Writing – review & editing, Visualization, Software, Formal analysis. SC: Writing – review & editing, Methodology, Formal analysis. MM: Writing – review & editing, Methodology, Formal analysis. PR: Writing – review & editing. PM: Writing – review & editing. BF: Writing – review & editing. ES: Writing – review & editing.

## Footnotes

^1^ https://github.com/biobakery/biobakery

^2^ https://cran.r-project.org/web/packages/tidyverse/index.html

^3^ https://metacyc.org/
